# Safety of bivalent live attenuated *Salmonella* vaccine and its protection against bacterial shedding and tissue invasion in layers challenged with *Salmonella*

**DOI:** 10.1016/j.psj.2022.101943

**Published:** 2022-04-30

**Authors:** Chen-Si Lin, Tsung-Lin Lu, Yi-An Chen, Hsin-Yi Yu, Chiu-Yi Wu, Wen-Yuan Yang

**Affiliations:** ⁎ Department of Veterinary Medicine, School of Veterinary Medicine, National Taiwan University, Taipei City, 106, Taiwan; † Zoonoses Research Center and School of Veterinary Medicine, National Taiwan University, Taipei City, 106, Taiwan; ‡ Elanco (Taiwan) Animal Health Co., Ltd. 9F, Taipei City, 105, Taiwan

**Keywords:** *Salmonella*, vaccine, safety, efficacy, layers

## Abstract

Nontyphoidal Salmonella infection was one of the predominant foodborne illnesses in humans. The medical burden and antimicrobial resistance of salmonellosis gained importance in public health and requested the poultry industry to seek effective measures to control the disease. The objective of this study was to evaluate the safety and effectiveness of a commercial bivalent live attenuated vaccine (AviPro Salmonella DUO) in specific-pathogen-free (**SPF**) chickens and field layers. It explored its safety and efficacy against medically important strains, Salmonella Enteritidis (**SE**) and S. Typhimurium (**ST**). The results demonstrated that ten vaccine doses in SPF chickens and regular doses in commercial layers showed desirable safety without affecting chicken health. Vaccinated layers demonstrated lower flock mortality and higher egg production performance than the unvaccinated layers during the raising and egg production periods. Additionally, no visceral colonization and egg contaminations were detected. Cloacal shedding of vaccine strains was noted, but the colonization of *Salmonella* disappeared within four weeks of the last vaccination. Regarding vaccine efficacy, one dose significantly reduced *Salmonella* cloacal shedding (*P* = 0.037 for SE and *P* = 0.027 for ST) and viable cell counts (*P* = 0.003 for SE and ST) on day 7 post the challenges. Significantly low *Salmonella* loads of cloacal samples on day 14 after the challenges were also determined in the vaccinated group (*P* = 0.006 for SE; *P* = 0.041 for ST). Triple immunizations effectively prevented layers from the cloacal shedding on either day 7 or day 14 post *Salmonella* challenges. Total viable counts of SE and ST in tissues of vaccinated layers were also reduced on day 14 after the challenges (*P* = 0.026 for SE; *P* = 0.002 for ST). To conclude, one dose of vaccine exhibited inhibitory effects on *Salmonella* shedding and tissue invasions in young layers. Following the regimen of triple vaccinations, *Salmonella* shedding was completely inhibited, and tissue invasions were significantly reduced. Incorporating this vaccine into a comprehensive *Salmonella* control program is promising to protect layers from the risks of contaminating the flocks and egg products.

## Introduction

Non–host-adapted *Salmonella* infections have gained importance in the poultry industry due to their role in the foodborne illness of human beings. The etiological agents of infections belong to *Salmonella enterica* subspecies *enterica* (Fuche et al., 2016). The costs from these foodborne illness in humans were estimated as 4 to 11 billion USD per year in the United States for medical care, loss of productivity, and premature deaths (Scharff, 2012). According to the results of epidemiological studies, more than 70% of *Salmonella* infections in humans have been associated with the consumption of poultry products, notably eggs and meat, contaminated with *Salmonella* Enteritidis (**SE**) and *S*. Typhimurium (**ST**) (Guo et al., 2011; Shah et al., 2017). These serovars regularly infected chickens without showing clinical symptoms. However, the threats of more prolonged shedding to contaminate chicken products and the environment drew substantial attention to prevent and control *salmonellosis* in the farms (Phillips and Opitz, 1995; Van Immerseel et al., 2005). Besides, the emergence of multi-antimicrobial resistance in *Salmonella* strains increased the difficulty of treating human patients suffering salmonellosis (Hall, 2010; Thung et al., 2016). As a result of adverse impacts on public health, the industry demands effective measures to control the prevalence and bacterial load of *Salmonella* in chicken flocks.

A control program including enhanced biosecurity, regular monitoring, and mass vaccination has been recognized as principal measures to reduce the prevalence of *Salmonella* on the farm (O'Brien, 2013; Poirier et al., 2008). It is of interest to note that several studies demonstrated the use of probiotics, prebiotics, or their combination (synbiotics) to ameliorate the gut information and bacterial colonization of SE or ST (Jazi et al., 2019; Khan and Chousalkar, 2020; Neveling et al., 2020; Price et al., 2020). Nonetheless, their effects varied in different studies because of different strains, administration protocols, or species. Up to date, the vaccine is considered an effective tool to control *Salmonella* infections in flocks. Live attenuated vaccines are broadly applied in layer chickens, whereas inactivated vaccines are regularly used in breeder chickens (Eeckhaut et al., 2018). The SE and ST were selected as targeted strains due to their association with recent outbreaks (Crouch et al., 2020). Live attenuated vaccines have the advantages of oral administration at any age, induction of cell-mediated portion of the protective immunity, and continuous passing vaccine strain among chickens (Desin et al., 2013). Nonetheless, the long persistence of live vaccine strain among chickens evoked the risk of contaminating chicken products and the concern for public health (Tan et al., 1997).

Theoretically, vaccines used to control *Salmonella* infection should be well-defined, safe, and effective for field applications (Van Immerseel et al., 2005; Barrow, 2007). For using live vaccines in layers, it is critical to understand the progress and impact of vaccine strains on production performance, environmental shedding, and egg contamination. A live vaccine requires a higher level of safety than other vaccines. This study evaluated the safety of a bivalent live attenuated vaccine targeting medically important *Salmonella* strains (SE and ST) in layers. Besides, we explored whether this vaccine conferred protection against bacterial shedding and tissue invasions in those chickens challenged with homologous serovars of *Salmonella* by qualitative and quantitative analyses.

## Materials and methods

### Vaccine

The commercial and lyophilized vaccine, AviPro Salmonella DUO (Elanco Animal Health, Greenfield, Indiana), was used in this study. It was a bivalent and live attenuated vaccine consisting of SE strain Sm24/Rif12/Ssq and ST strain Nal2/Rif9/Rtt. Each dose contained a final concentration of SE and ST between 1 × 10^8^ and 6 × 10^8^ colony forming units (**CFU**). Both SE and ST strains were sensitive to erythromycin. The SE stain was further identified as resistant to streptomycin and rifampicin. In contrast, ST was resistant to nalidixic acid and rifampicin. Before the experiments, vaccines were examined by laboratory tests, including characteristics, purity, vacuum, humidity, viable cell count, serotyping, and vaccine strain validation tests.

### Salmonella Challenge Strains and Inoculum Preparation

The SE strain 147 Nal^res^ and ST strain 9098 Nal^res^ provided by Elanco Animal Health Co., Ltd. (Elanco Animal Health, Greenfield, Indiana) were used as *Salmonella* challenge strains. Each vial contained the challenge strain with a bacterial cell count of 3.2 × 10^10^ CFU/mL. The viable cell counts in vials were validated by direct cultures on xylose lysine deoxycholate (**XLD**) agar (Sigma, St. Louis, Missouri, USA) plates with serial dilutions. Plate agglutinations were used to confirm the serotypes of challenge strains with antisera (O9+ and Hg+, Hm+ for SE; O5+ and Hi+, H1- for ST). Oral inocula for *Salmonella* challenges were prepared to the concentration of 10^9^ CFU per chicken by diluting the original stock ten times with PBS solution (Sigma, St. Louis, Missouri).

### Microbiological Isolation and Analysis

Bacterial isolations for *Salmonella* in the swab, fecal, pooled visceral (liver, spleen, cecum, ovary, and fallopian tube), and pooled tissue (liver, spleen, and cecum) samples from two trials were conducted following ISO 6579:2002 (Microbiology of food and animal feeding stuffs-Horizontal method for the detection of *Salmonella* spp.). Plate agglutinations were used for serotyping *Salmonella* strains or isolates with antisera to O and H antigens. The SE was identified by positive reactions to O9 and Hg, Hm. ST was determined while the reactions showed positive for O5 and Hi and negative for H1.

The AviPro Plate (Elanco Animal Health, Greenfield, Indiana) was applied to differentiate vaccine and challenge strains. The minimal inhibitory concentration (MIC) breakpoints were used to determine the in vitro antimicrobial activities of rifampicin and streptomycin. A single colony was selected and placed in 0.9% normal saline with turbidity similar to a 0.5 McFarland standard. Then, a volume of 50 μL of the bacterial solution was placed into 10 mL of Muller-Hinton broth (Sigma, St. Louis, Missouri) and mixed thoroughly, and a 100 μL of broth was drawn in the microwell of the AviPro Plate. The plate was sealed and incubated at 35 to 37°C for 18 to 24 h. The results of the AviPro Plate were interpreted visually by the following criteria. The SE could grow in microwells containing rifampicin and streptomycin was a vaccine strain. In contrast, the ST vaccine strain grew in microwells containing rifampicin but failed to multiply in microwells with streptomycin. Both SE and ST challenge strains were sensitive to these two antibiotics. Hence, they did not grow in microwells containing either rifampicin or streptomycin.

### Experimental Designs and Treatments

The safety and efficacy of AviPro Salmonella DUO vaccines in layers were evaluated, followed by single and triple vaccinations in specific-pathogen-free (**SPF**) chickens and commercial layers. These layers were included to investigate the effects of vaccines under the disease-free status and approximately the field condition. The safety evaluation in SPF chickens and the efficacy against *Salmonella* challenges were performed in the animal biosafety level (**ABSL**)-2 poultry facility at the Animal Resource Center of National Taiwan University (**NTU**).

#### SPF Chicken Trial

The safety of the vaccine was investigated in a group of 10 one-day-old SPF chicks orally administered with 10 doses of vaccine (0.5 mL). During the monitoring period of three weeks, all chicks should be alive without any clinical symptoms. For the evaluation of vaccine efficacy, 48 one-day-old SPF chicks were randomly allotted into 4 groups (12 chicks per group), designated as SPF-DUO-SE, SPF-DUO-ST, SPF-SE, and SPF-ST, as summarized in Table 1. Groups of SPF-DUO-SE and SPF-DUO-ST were orally inoculated with one dose of vaccine (0.03 mL) at the age of day 2 (vaccinated groups). Groups of SPF-SE and SPF-ST were contrarily treated with the placebo (unvaccinated groups). *Salmonella* challenges were performed in each SPF chicken on day 14 after vaccinations or placebo treatments in a blinded manner. The SE 147 Nal^res^ was applied to SPF-DUO-SE and SPF-SE groups, and ST 9098 Nal^res^ were given for challenges to SPF-DUO-ST and SPF-ST groups. Chickens were euthanized by CO_2_ on day 14 after the *Salmonella* challenges. Cloacal swabs were collected on days 7 and 14 after the challenges to detecting the prevalence of bacterial shedding. The 1.5 gm of the liver, spleen, and cecum tissues per chicken were collected 14 d post the challenges to investigate the prevalence of tissue invasion. Quantitative measurements of *Salmonella* cell loads were conducted in those swabs and homogenates from tissue samples (1 gm per sample) in Tetrathionate-Brilliant-green bile enrichment (TBG) broth (Sigma, St. Louis, Missouri). The mixtures were diluted through serial dilutions and placed in brilliant-green phenol-red lactose sucrose (**BPLS**) agar (Sigma, St. Louis, Missouri) plates containing 100 μg/mL of nalidixic acid (Sigma, St. Louis, Missouri). After the cultivation at 37°C for 18 to 24 h, the viable *Salmonella* cell count was calculated. *Salmonella* colonies were further serotyped by the methodology described in the previous section. Antibiotics were not provided during the whole trial.

**Table 1:** The SPF chicken trial and field chicken trial designs in the ABSL-2 poultry facility.

Trial	Group	N	AviPro Salmonella DUO	*Salmonella* challenge		Cloacal sampling (dpc)	Tissues sampling (dpc)
				Strains	Time (dpv)		
SPF chickens	SPF-DUO-SE	12	Vaccinated*	SE 147 Nal^res^	14	7, 14	14
	SPF-SE	12	Non-vaccinated	SE 147 Nal^res^	14	7, 14	14
	SPF-DUO-ST	12	Vaccinated*	ST 9098 Nal^res^	14	7, 14	14
	SPF-ST	12	Non-vaccinated	ST 9098 Nal^res^	14	7, 14	14
Commercial layers	DUO-SE^a^	12	Vaccinated#	SE 147 Nal^res^	14	7, 14	14
	SE	12	Non-vaccinated	SE 147 Nal^res^	14	7, 14	14
	DUO-ST	12	Vaccinated#	ST 9098 Nal^res^	14	7, 14	14
	ST	12	Non-vaccinated	ST 9098 Nal^res^	14	7, 14	14
	Control	12	Non-vaccinated	-	-	7, 14	14

The upper index of

⁎ indicates that one dose of vaccine was used at the age of day 2.

# represents triple vaccinations applied at the age of day 5, week 8, and week 18. N = numbers of sample; dpv: days post-vaccination; dpc: days post-challenge. Tissues samples in this table include liver, spleen, and cecum.

a one dead chicken was found in the Filed-DUO-SE group on day five post-challenge. The chicken was sent to the necropsy and culture of the causative agent. The results of histopathology showed *E. coli* infection with yolk peritonitis. Bacterial cultures by *Salmonella-specific* culture media (TBG and BPLS) showed the negative result for *Salmonella*.

#### Commercial Layers Trial

A commercial layer farm starting with 8,320 one-day-old chicks was included in this trial. Before introducing day-old chicks, each chicken house was monitored for *Salmonella* by randomly collecting 24 swab samples (12 swabs from 2 pairs of shoes and boots; 12 swabs from cages). On arriving, 12 transportation boxes were randomly selected to collect 24 chicks and 12 padding samples (2 chicks and 1 padding sample per box) for detecting *Salmonella*. Dead chicks during the transportation were also collected for detection. Meanwhile, day-old chicks were randomly assigned to the vaccinated and unvaccinated groups for the following treatments. The introduced chicks and the raising environment were confirmed to be *Salmonella*-free. Chickens were raised without restraint to the feed and drinking water during the whole trial. Following the field husbandry, chickens were transferred to pullet houses and egg-laying houses from the age of weeks 7 to 15 and weeks 16 to 35. Two weeks before leaving the pullet houses, 2 pairs of boot samples per house were collected and tested for *Salmonella* status. Twelve swabs from cages and eight scrape samples from the surface of the wooden strips per laying house were tested for *Salmonella* prior to moving into egg-laying houses. According to the manufacturer's instructions, triple vaccinations were conducted at the age of day 5, week 8, and week 18 with a regular dose of vaccine (0.03 mL/chicken).

In this trial, chicken health, flock mortality, production performance by egg production rate (**EPR**), bacterial shedding, tissue colonization, and egg contaminations by vaccine strains were investigated on the subject of vaccine safety in field applications. Clinical symptoms relative to salmonellosis were monitored and recorded on a daily basis. Flock mortality was documented and compared the difference between groups during the brooding (weeks 1–6), pullet (weeks 7–15), and egg-lying (weeks 16–35) periods. The EPR was collected at an interval of 5 wk, starting from the age of week 16 to week 35. During the trial period, 20 cloacal swabs in each group were collected 2 d after the first, second, and third vaccinations and 7, 14, 21, 28, 35, 42 d post the last vaccination to depict the shedding progress of vaccine strains. At the age of weeks 25, 30, and 35, 20 chickens in each group were randomly selected and euthanized. Their livers, spleens, ceca, ovaries, and fallopian tubes were collected to monitor vaccine strains' tissue colonization. To evaluate the potential of egg contamination, 50 eggs were collected from the vaccinated and unvaccinated groups every 4 wk starting from week 19. Eggshell and content samples were subjected to *Salmonella* isolation following the official methodology from the Ministry of Health and Welfare in Taiwan (MOHWM0025.01).

As for assessing the vaccine efficacy against *Salmonella* challenges, 60 chickens (24 and 36 chickens from the vaccinated and unvaccinated groups, respectively) at the age of week 25 were randomly selected from the egg-laying houses and transferred to the ABSL-2 animal facility. After that, they were allotted into 5 groups, such as DUO-SE, DUO-ST, SE, ST, and Control groups (n = 12 per group), by randomization. The trial design was summarized in Table 1. Chickens within DUO-SE and SE groups were vaccinated in the field and challenged with SE 147 Nal^res^ in the ABSL-2 facility. In contrast, chickens in DUO-ST, ST, and Control groups were not vaccinated. DUO-ST and ST groups were challenged with ST 9098 Nal^res^, whereas the Control group served as the negative control. Before *Salmonella* challenges, cloacal swabs were collected from all chickens to confirm their *Salmonella*-free status. Chickens were euthanized by CO_2_ on day 14 after the *Salmonella* challenges. Cloacal swabs were collected on days 7 and 14 after the challenges to detecting the prevalence of bacterial shedding. The 1.5 gm of the liver, spleen, and cecum tissues per chicken were collected 14 d post the challenges to investigate the prevalence of tissue invasion. Quantitative measurements of *Salmonella* cell loads were also conducted in those swabs and homogenates from tissue samples (1 gm per sample). Calculation of *Salmonella* cell count and serotyping of *Salmonella* colonies were performed using the same methodologies described in the previous sections.

### Monitoring and Vaccine Immunization

A veterinary inspector without the perception of treatments was assigned to monitor chickens' health in 2 trials daily. Any chickens that died during the trials were sent to an impartial third-party unit (Graduate Institute of Molecular and Comparative Pathobiology, School of Veterinary Medicine, NTU) for pathological examinations to confirm the cause of death. Flock mortality and EPR were recorded and documented by the owner in the field. The technician from the Elanco company conducted the examinations of the water supply system for free-flowing and immunizations of vaccines. The success of vaccine delivery was validated by using AviBlue (Elanco Animal Health, Greenfield, Indiana).

### Ethical Approval

The challenges in chickens and protocols were approved by National Taiwan University Institutional Animal Care and Use Committee (NTU-109-EL-00115). Chicken distress was minimized during the operations. Euthanasia was performed on day 14 post the *Salmonella* challenges by CO_2_ asphyxiation.

### Statistical Analysis

Statistical significance was calculated utilizing SAS software version 9.4 (SAS Institute, Inc., Cary, NC). The difference in flock mortality and EPR between the vaccinated and unvaccinated groups were compared using the Chi-squared (χ2) test. Fisher's exact test compared the prevalence of *Salmonella* shedding and tissue invasions between groups in 2 trials. Visible cell counts were converted into a logarithmic form for comparisons. The Wilcoxon Rank-Sum test determined the significant difference in *Salmonella* cell counts between groups. Statements of statistical significance were based on the level of *P* ≤ 0.05.

## Results

### Quality of Vaccine

Upon gross observation, the vaccine exhibited inherent physical properties. The vials were white lyophilized homogeneous powder without abnormal odors and free from foreign matter by the component analysis. The reconstituted vaccine solution with 10 mL of sterile purified water was cultivated on blood, nutrient, BPLS, Sabouraud, and Gassner agar for three days. No bacteria other than the *Salmonella* vaccine strains were detected, and the serovars were further confirmed as SE and ST by serotyping. The AviPro Plate results of those serovars showed antimicrobial activities consistent with vaccine strains. On the other hand, electrodeless discharge was performed with Tesla Coil within 5 mm of a darkroom. The result of a positive reaction (discharge) showed that the vial was in a vacuum. The humidity of powder in vaccines was measured as 0.045% to 0.047% by the Karl-Fisher method, meeting the requirement of being below 4% (inclusive). Lastly, the concentration of SE and ST strains in tested vaccines counted from 3 replicates demonstrated as 3.2 to 5.0 × 10^8^ CFU/dose and 4.2 to 5.2 × 10^8^ CFU/dose, conforming to the strain number per dose (above 1 × 10^8^ CFU/dose per strain) described in the package insert.

### Salmonella-Free Status of Chickens and Environments

The dead and live chicken samples during the period of transportation and introduction were tested negative for *Salmonella*. Swab samples collected from the transportation boxes, pullet houses, and egg-laying houses were also tested negative for the isolation. Fecal samples collected from egg-laying houses at the chicken age of weeks 15, 25, and 35 were *Salmonella*-free by bacterial isolations. The results were summarized in Table 2, highlighting that the chicks, transportation boxes, and chicken houses in the field trial had not been subject to previous *Salmonella* infection and environmental contamination. During the raising periods in chicken houses, the flocks still maintained the *Salmonella*-free status.

**Table 2:** Monitoring results of chickens and environments for *Salmonella* in field chicken trial.

Site	Sampling time	Samples	Bacterial isolation
Pullet houses	Before the introduction of chicks	Swabs of two pairs of shoes and boots per house	Negative
		12 swabs from cages of each house	Negative
	During the introduction of chicks	24 live chicks from transportation boxes (two for each box)	Negative
		12 padding samples from transportation boxes	Negative
		Dead chicks during the transportation	Negative
	2 weeks before leaving	Swabs of two pairs of shoes and boots per house	Negative
Egg-laying houses	Before the introduction of pullets	12 swabs from cages of each house	Negative
		Scrape samples on the surface of the wooden strips at each house	Negative
	At the age of weeks 15, 25, and 35	2 fecal samples (150g/sample) from the wooden strips carrying feces at each house	Negative

The samples described in the table were collected randomly, including swabs, transportation boxes, live chicks, padding samples, scrape samples, and fecal samples.

### Effects of Vaccine Strains on Chicken Health, Flock Mortality, and EPR

After receiving ten doses of vaccine at one time, immunized SPF chicks were shown in healthy condition without mortality and clinical symptoms of *Salmonella* infections during the observation period of 3 wk. For the applications of standard vaccine dose in commercial layers, they did not exhibit clinical infestations of salmonellosis after the first, second, and third vaccinations. On the subject of flock mortality in the field, the vaccinated group presented significantly low mortality during the pullet (*P* = 0.003) and egg-laying (*P* = 0.004) periods compared to the unvaccinated group (Table 3). The application of AviPro Salmonella DUO by triple vaccination regimen did not compromise the EPR in the field. Additionally, the higher EPR in vaccinated chickens was noted during the egg-laying period compared to the unvaccinated. Significant differences appeared during weeks 16 to 20 and 26 to 30 (*P* = 0.013 and 0.024). However, the overall EPR between groups during weeks 16 to 35 was not significantly different (*P* = 0.165).

**Table 3:** Parameters applied to evaluate AviPro Salmonella DUO's safety and their results in the field chicken trial.

Parameters	Vaccinated group		Unvaccinated group		*P* value
	SE	ST	SE	ST	
*Mortality*					
Weeks 1–6	0.6%		0.5%		0.887
Weeks 7–15	0.5%		1.3%		0.003*
Weeks 16–35	5.8%		7.6%		0.004*
*Egg production rate (EPR)*					
Weeks 16–20	42.5%		39.2%		0.013*
Weeks 21–25	84.2%		82.6%		0.117
Weeks 26–30	88.4%		86.3%		0.024*
Weeks 31–35	81.8%		79.8%		0.069
Weeks 16–35	82.8%		81.3%		0.165
*Vaccine strain shedding*					
Visceral samples (N = 20)					
Weeks 25	0%	0%	0%	0%	–
Weeks 30	0%	0%	0%	0%	–
Weeks 35	0%	0%	0%	0%	–
Cloacal swabs (N = 20)					
First vaccination					
2 dpv	0%	0%	0%	0%	–
Second vaccination					
2 dpv	5%	10%	0%	0%	–
Third vaccination					
2 dpv	15%	25%	0%	0%	–
7 dpv	40%	25%	0%	0%	–
14 dpv	15%	5%	0%	0%	–
21 dpv	10%	10%	0%	0%	–
28 dpv	0%	0%	0%	0%	–
35 dpv	0%	0%	0%	0%	–
42 dpv	0%	0%	0%	0%	–
Egg samples (N = 50)					
Weeks 19	0%	0%	0%	0%	–
Weeks 23	0%	0%	0%	0%	–
Weeks 27	0%	0%	0%	0%	–
Weeks 31	0%	0%	0%	0%	–
Weeks 35	0%	0%	0%	0%	–

SE: *S*. Enteritidis; ST: *S*. Typhimurium; N = numbers of sample;

⁎ : *P < 0.05* by Chi-square test; dpv: days-post vaccination. Visceral samples in this table include liver, spleen, cecum, ovary, and fallopian tube. The egg sample included the swabs on the eggshell and egg contents. -: statistical analysis was not performed.

### Shedding Progress of Vaccine Strains and Their Ability to Colonize Tissues and Contaminate Eggs

The results are summarized in Table 3. The first vaccination of AviPro Salmonella DUO did not shed vaccine strains on day 2 after the immunization. However, the onset of cloacal shedding was observed on day 2 after the second and third vaccinations. Triple vaccinations demonstrated a higher bacterial shedding rate in the vaccinated group. Shedding progress revealed that the shedding rate continuously decreased from day 7 post last vaccination, and the cloacal shedding ceased on day 28 after the last vaccination. In contrast to bacterial shedding through the cloaca, vaccine strains did not colonize tissues, including livers, spleens, ceca, ovaries, and fallopian tubes, collected from the vaccinated chickens at the age of weeks 25, 30, and 35. The *Salmonella*-negative results of multiple egg collections showed no contamination of vaccine strains either on eggshells or in egg contents at the age of weeks 19, 23, 27, 31, and 35.

### Vaccine Efficacy Against Bacterial Shedding and Tissue Invasions by SE and ST Challenges

The results of the qualitative analysis showed that one vaccination significantly reduced the prevalence of cloacal shedding in SPF chickens (*P* = 0.037 for SE; *P* = 0.027 for ST) on day 7 after the *Salmonella* challenges, as summarized in Table 4. The prevalence of bacterial shedding and tissue invasion in the vaccinated group on day 14 after *Salmonella* challenges was lower than in the unvaccinated group. However, the differences were not significant. Triple immunizations effectively prevented commercial layers from the cloacal shedding (zero prevalence) on either day 7 or day 14 after *Salmonella* challenges. The prevalence of ST tissue invasions was significantly reduced in vaccinated layers compared to the unvaccinated on day 14 after the challenges (*P* < 0.001). Although the prevalence of SE invasions was also reduced in vaccinated field chickens, a significant difference was not detected (*P* = 0.069).

**Table 4:** Prevalence of *Salmonella* in cloacal and tissue samples from the vaccinated and unvaccinated groups after the challenges.

Challenge serovar	Time and samples	Vaccinated group			Unvaccinated group			*P* value
		N	+	Prevalence	N	+	Prevalence	
*SPF chickens*								
SE	7 dpc							
	Swab	12	7	58.3%	12	12	100.0%	0.037*
	14 dpc							
	Swab	12	8	66.7%	12	12	100.0%	0.093
	Tissue	12	3	25.0%	12	10	83.3%	0.012*
ST	7 dpc							
	Swab	12	5	41.7%	12	11	91.7%	0.027*
	14 dpc							
	Swab	12	3	25.0%	12	8	66.7%	0.100
	Tissue	12	2	16.7%	12	6	50.0%	0.193
*Commercial layers*								
SE	7 dpc							
	Tissue	11	0	0.0%	12	2	16.7%	0.478
	14 dpc							
	Swab	11	0	0.0%	12	3	25.0%	0.217
	Tissue	11	1	9.1%	12	6	50.0%	0.069
ST	7 dpc							
	Tissue	12	0	0.0%	12	1	8.3%	1.000
	14 dpc							
	Swab	12	0	0.0%	12	1	8.3%	1.000
	Tissue	12	1	8.3%	12	10	83.3%	<0.001*

SE: *S*. Enteritidis; ST: *S*. Typhimurium; N = numbers of sample;

+ : positive for the isolation of *Salmonella*;

⁎ *P < 0.05* by Fisher exact test; dpc: days post-challenge. Tissue in this table was pooled samples, including the liver, spleen, and cecum.

The quantitative data of bacterial loads revealed that one dose of vaccination could significantly reduce viable cell counts of SE and ST in cloacal samples collected from SPF chickens on day 7 (*P* = 0.003 for both SE and ST) and day 14 (*P* = 0.006 for SE; *P* = 0.041 for ST) after *Salmonella* challenges. The results are summarized in Table 5. Besides, one time of immunization also significantly decreased the cell load of SE in the tissues (*P* = 0.005) collected from SPF chickens on day 14 after SE challenges. When the triple vaccinations were conducted, cloacal shedding was thoroughly prevented in field chickens on either day 7 or day 14 post *Salmonella* challenges. Moreover, total viable counts of SE and ST in these vaccinated chickens were significantly reduced on day 14 after the challenges (*P* = 0.026 for SE; *P* = 0.002 for ST). Accordingly, one dose of vaccine exhibited inhibitory effects on *Salmonella* shedding and tissue invasions in young layers. Following the regimen of triple vaccinations, *Salmonella* shedding and tissue invasions were effectively inhibited and significantly reduced.

**Table 5:** Total viable counts (mean log_10_ CFU/g) of *Salmonella* in cloacal and tissue samples from the vaccinated and unvaccinated groups after the challenges.

Challenge serovar	Time and samples	Vaccinated group	Unvaccinated group	*P* value
*SPF chickens*				
SE	7 dpc			
	Swab	2.347	4.424	0.003*
	14 dpc			
	Swab	2.522	5.164	0.006*
	Tissue	0.625	2.567	0.005*
ST	7 dpc			
	Swab	1.350	3.653	0.027*
	14 dpc			
	Swab	1.118	3.175	0.041*
	Tissue	0.162	0.687	0.053
*Commercial layers*				
SE	7 dpc			
	Swab	0.000	0.354	0.186
	14 dpc			
	Swab	0.000	0.759	0.093
	Tissue	0.127	1.843	0.026*
ST	7 dpc			
	Swab	0.000	0.204	0.359
	14 dpc			
	Swab	0.000	0.232	0.359
	Tissue	0.426	2.359	0.002*

N = numbers of sample;

⁎ : *P < 0.05* by Wilcoxon Rank-Sum test; dpc: days post-challenge. Tissue in this table was pooled samples, including the liver, spleen, and cecum.

## Discussion

A multi-country stochastic study on *Salmonella* source attribution showed that layers were the leading reservoir for human salmonellosis in the European countries (LV et al., 2015). The SE and ST were demonstrated as the most dominant serovars in these foodborne outbreaks (EFSA & ECDC, 2018). They regularly infected layers through horizontal transmissions to colonize the ceca and invade their livers and spleens. Consequently, affected chickens became life-long carriers and the source of contamination to eggs (Hu et al., 2021). It is noteworthy that SE dominated the egg-associated *Salmonella* outbreaks (Patrick et al., 2004) because of their supplementary ability to colonize the ovary and oviduct, leading to in-egg contaminations (Gast et al., 2013). On the other hand, ST was reported to cause severe diseases in very young chickens, contributing to public health issues and significant economic losses (Hesse et al., 2018). Based on the recent rise of those zoonotic *Salmonella* infections in layers, a safe and effective prophylactic that protects them from bacterial shedding and organ invasions by pathogenic *Salmonella* without compromising the regular productions was demanded. While the actual prevalence of *Salmonella* infections in laying hens remained little investigated or published, the predominance of SE and ST in foodborne cases elicited the necessity to implement effective measures against these serovars.

Ten doses of vaccine in SPF chickens and regular doses in commercial layers showed desirable safety within the short-term period (3 wk) and long-term applications (35 wk) without affecting chicken health. Ordinarily, vaccine operations may accompany a small number of deaths and compromise some performance due to stress. Layers with triple vaccinations showed lower flock mortality and higher egg production performance. Further studies are recommended to investigate the underlying mechanisms. In this study, the shedding time of vaccine strains was consistent with Hahn's findings that they could be detected up to 21 d after the vaccination (Hahn and Vielitz, 2000). However, another study using a single SE strain demonstrated a different result that cloacal shedding ceased 5 to 10 d after the first and multiple vaccinations (Huberman et al., 2019). The longer shedding duration of this bivalent vaccine may result from the interactions between SE and ST strains or the environmental difference between well-controlled facilities in Huberman's study and field premises in our study that contributed to more stress. Under stress, it was well-documented to induce bacterial shedding of ST in feces (Traub-Dargatz et al., 2006). Since immunosuppression from laying stress may promote the appearance of vaccine strains, egg samples were collected and monitored at the onset of laying. The *Salmonella*-free results of multiple egg collections showed that vaccine strains did not possess the ability as the pathogenic strains to contaminate eggs through the route of vertical and fecal transmissions. Long persistence in tissues was conceived to induce protective immune responses by live attenuated vaccines. The negative results for *Salmonella* revealed that these vaccine strains could not colonize and invade livers, spleen, ovaries, and oviducts, which the wild type of *Salmonella* most frequently targeted (Theuß et al., 2018). The stimuli of vaccine stains may have been sufficient for well-established adaptive immunity before the time of visceral sample collections (week 25). Based on the evidence on cloacal shedding, the vaccine strains were cleared from vaccinated layers within 4 wk of administration (week 22), elucidating the possible mechanism for *Salmonella*-free results of visceral samples collected from weeks 25 to 35. Overall, these vaccine strains were recognized as harmless in field applications, and their presence in cloaca at the age of weeks 19 to 21 did not contaminate eggs collected at the age of week 19.

In the present study, one dose vaccine elicited significant effects against the shedding of SE and ST on day 7 post their challenges. These effects on day-old chicks may result from the colonization-inhibition induced by the vaccine strains (Desin et al., 2013). It provided rapid protection before the chicks could exert immunity against vaccine strains. This colonization-inhibition was most efficacious in the homogenous serovars (Eeckhaut et al., 2018) but ineffective in different serotypes (Methner et al., 2010; Methner et al., 2011). Although one dose of vaccine could counteract the cloacal shedding and tissue invasions from *Salmonella* challenges till day 14, the protection was not statistically significant. Protective effects induced by the vaccination mainly depended on the strength of host adaptive immunity (Desin et al., 2013). Booster vaccinations were regularly required for a more effective adaptive immune response to stimulate cellular and humoral immunity. In the field applications, triple vaccinations at the age of day 2, week 8, and week 18 thoroughly prevented the cloacal *Salmonella* shedding on day 7 and day 14 post the challenges. Above 90% of vaccinated chickens were free from tissue invasions by *Salmonella*, significantly for ST. These results provided an effective immunization scheme for standard practice in the layer industry to prevent the transmissions of SE and ST.

The challenge models of SE and ST were verified by examining the bacterial shedding in SPF chickens after the inoculations. Ninety-two to a hundred percent of unvaccinated SPF chickens shed challenge strains through their cloaca. Nevertheless, *Salmonella* challenges in commercial layers did not demonstrate the high prevalence of cloacal shedding in the unvaccinated layers as SPF chickens presented. Gut microbiota has been demonstrated to influence the intestinal morphology, nutrient absorption, immune regulations, and bacterial pathogenesis of enteric diseases (Khan et al., 2020; Kogut et al., 2020). The intestinal microflora was recognized to play a role in the colonization of the intestines by pathogens (Kempf et al., 2020; Mead, 2000). A recent study demonstrated that feeding newly-hatched chickens with microbiota from 3-wk-old or older chickens protected them against SE challenges (Varmuzova et al., 2016). The evidence indicates that commercial layers may possess diverse microflora, further conferring the colonization resistance to SE/ST and low shedding prevalence in unvaccinated layers. Further investigations addressing the gut microbiota may provide insights into this phenomenon. A growing number of studies recently targeted gut microbiota to explore the probiotic and prebiotic in feed to promote the colonization resistance by flora, subsequently decreasing the colonization and adverse effects of *Salmonella* (Smialek et al., 2019; Neveling et al., 2020; Zhang et al., 2021).

*Salmonella* loads in the ceca of vaccinated chickens after the challenges were quantified and compared to the unvaccinated. The reduction of *Salmonella* loads in layers aims to eventually diminish or prevent the risks of foodborne outbreaks in human beings. Our results demonstrated that one vaccination significantly decreased *Salmonella* loads in the cloaca. When triple vaccinations were implemented, *Salmonella* was completely eliminated from the cloaca. At the same time, live *Salmonella* cells in the liver, spleen, and ceca of vaccinated chickens were significantly diminished. One dose of vaccine was shown insufficient to exert a profound effect against ST invasions, indicating ST might require a more persistent expression of antigens to the host immune system compared to SE. The significant reduction of colonized *Salmonella* cell number in tissues was crucial for preventing and controlling salmonellosis on the farm and at the abattoir (Buncic and Sofos, 2012; Foods, 2019), further lowering the contamination of poultry products and the incidence of human cases. The efficacy of this live *Salmonella* vaccine demonstrated the desirable protections for layers in the field.

In conclusion, the field applications of vaccinal SE and ST neither exhibited adverse effects on chicken health and flock mortality nor resulted in egg contaminations. This bivalent live attenuated vaccine provided safe and efficacious mechanisms to confer protection against cloacal shedding and organ invasions after the infections of homologous *Salmonella* serovars. Incorporating this vaccine into a comprehensive *Salmonella* control program is promising to protect layers from the risks of contaminating the flocks and egg products.

## Acknowledgments

The authors would like to thank the animal-keeping facility and laboratory personnel for their assistance on chicken care, *Salmonella* challenges, and logistics.

## Disclosures

The authors declare the following financial interests, which might be considered as potential competing interests: the work was funded by Elanco Animal Health, which has commercialized the vaccine used in this study. Some authors are employees of Elanco (Taiwan) Animal Health.
